# Differential molecular characterization of human papillomavirus‐associated oropharyngeal squamous cell carcinoma and its prognostic value

**DOI:** 10.1111/jcmm.70073

**Published:** 2024-10-13

**Authors:** Huanhuan Wang, Qihe Zhang, Zhuangzhuang Zheng, Ying Xin, Xin Jiang

**Affiliations:** ^1^ Jilin Provincial Key Laboratory of Radiation Oncology and Therapy, The First Hospital of Jilin University and College of Basic Medical Science Jilin University Changchun China; ^2^ Department of Radiation Oncology The First Hospital of Jilin University Changchun China; ^3^ NHC Key Laboratory of Radiobiology, School of Public Health Jilin University Changchun China; ^4^ Key Laboratory of Pathobiology, Ministry of Education, College of Basic Medical Science Jilin University Changchun China

**Keywords:** human papillomavirus, oropharyngeal squamous cell carcinoma, overall survival, prognostic factors, progression‐free survival

## Abstract

Human papillomavirus (HPV) infection is a causative factor in the occurrence and progression of oropharyngeal squamous cell carcinoma (OPSCC). In recent years, clinical studies have found that HPV‐positive OPSCC patients may present a better prognosis than HPV‐negative patients, yet the underlying causes are unclear. This study aimed to investigate the relevance of HPV infection and the prognosis of OPSCC. On this basis, we aimed to establish a prediction model to accurately predict the prognosis and guide clinical practice. We analysed the records of 233 patients with OPSCC. Cox regression was applied to identify factors associated with survival. Moreover, variables with significant discrepancies were integrated into a nomogram model to predict prognosis. The results showed that HPV was an independent prognostic factor for OS and PFS. Immunoglobulin Heavy Constant Mu (*IGHM*) mRNA was significantly upregulated in patients with HPV‐positive OPSCC. Crucially, *IGHM* expression was associated with better prognosis. The receiver operating characteristic (ROC) curves, calibration curves, and decision curve analysis both confirmed that the prognostic model exhibits good performance. In summary, HPV infection were independent prognostic factors for OPSCC. IGHM may be the key contributors to the prognostic differences in HPV‐associated OPSCC. This nomogram model was able to accurately predict the prognosis of patients.

## 1 INTRODUCTION

Oropharyngeal squamous cell carcinoma (OPSCC) is a common malignancy of the head and neck.^1^ OPSCC is a heterogeneous tumour covering the posterior pharyngeal wall, the soft palate, the tonsils and the root of the tongue. The tonsils and the root of the tongue are the most common anatomical sites, accounting for 96% of all cases.^2^ Globally, OPSCC is a major public health problem, with approximately 98,000 new cases and 48,000 deaths per year.^3^ Previously, the primary treatment for OPSCC was the combination of surgery, radiotherapy and chemotherapy. In recent years, despite the widespread use of novel treatments, such as targeted therapies, immunotherapies and anti‐angiogenic therapies, the 5‐year overall survival (OS) of patients with OPSCC has been approximately 50%, and failed to improve significantly over the past 20 years.^4^, ^5^ Owing to the high heterogeneity of OPSCC at the cellular and molecular level, it is difficult to achieve satisfactory anti‐tumour effects. Therefore, screening for sensitive molecular markers that predict patient prognosis and identifying effective molecular targets to improve prognosis is the focus of research worldwide.

It has been reported that smoking and alcohol consumption are common risk factors for the occurrence and progression of OPSCC.^6^ However, in recent years, the incidence of human papillomavirus (HPV) infection‐associated OPSCC has increased,^7^ accounting for 71% and 51.8% of OPSCC in the United States and the United Kingdom, respectively.^8^, ^9^ HPV is a double stranded, circular cyclic DNA virus, of which more than 200 types have been identified and classified into high‐ and low‐risk types.^10^ HPV16 and HPV18 are the two most common high‐risk HPV types in OPSCC,^11^ and E6 and E7 are the two main oncogenes.^12^ HPV inactivates the retinoblastoma tumour suppressor protein (Rb), which upregulates the expression of p16INK4a (p16).^13^ The College of American Pathologists guidelines recommend that immunohistochemical staining (IHC) to detect p16 expression can be an effective method to diagnose HPV infection.^14^, ^15^, ^16^

Considering the different populations, tumours, and molecular characteristics of HPV‐positive and ‐negative OPSCC, the latest version of the American Joint Committee on Cancer (AJCC) staging system distinguishes between HPV‐positive and HPV‐negative OPSCC.^17^ Patients with HPV‐positive OPSCC have a better prognosis and survival compared with HPV‐negative patients.^18^, ^19^ In a study of 58 patients with OPSCC treated with postoperative radiotherapy for 5.8 years, patients with HPV‐positive OPSCC had lower tumour recurrence rates and better OS and progression‐free survival (PFS).^18^ Another study of 465 patients with stage III/IV OPSCC treated with concurrent radiotherapy showed that patients with HPV‐positive OPSCC had a 2‐year OS of 91% and a PFS of 87%, which were significantly higher than those in the HPV‐negative group.^19^ Therefore, the effect of HPV infection on the prognosis of patients with OPSCC and its underlying molecular mechanisms need to be further investigated.

This study aims to investigate the role of HPV infection in determining the prognosis of OPSCC, and to identify the key genes contributing to molecular differences in tumour characteristics. On this basis, we aim to establish a prediction model to accurately predict the prognosis of OPSCC.

## 2 MATERIALS AND METHODS

### 2.1 Patients

We identified 233 eligible patients with OPSCC who were treated at the First Hospital of Jilin University between January 2010 and January 2023. This retrospective study was approved by the Ethics Committee of the First Hospital of Jilin University. Since this was a retrospective study, consent was not required for inclusion in the analysis. The inclusion criteria were: (1) OPSCC diagnosed by histopathology; (2) complete clinical information; (3) complete follow‐up data; and (4) that the patient received a treatment regimen the patient received was the standard regimen recommended by the National Comprehensive Cancer Network guidelines. The exclusion criteria were: (1) distant metastasis or other malignancies before this study; (2) serious life‐threatening diseases such as serious cardiovascular, respiratory, or kidney disease; (3) non‐completion of the treatment plan; and (4) incomplete survival data. Patients were staged according to the AJCC cancer staging manual, 8th/7th edition.^20^ We collected data on the following prognostic factors: gender, age, alcohol and tobacco consumption, site, pathological differentiation, clinical stage, lymphovascular invasion, perineural invasion, surgical margin, history of diabetes and related IHC indicators.

Data on the survival, disease progression, recurrence and death status of the selected patients were summarized at 3‐month intervals. PFS is defined as the time from diagnosis to disease progression, death (if no progression was reported before death), or the last follow‐up. OS is defined as the time from diagnosis to death or the last follow‐up.

### 2.2 Database analysis

The Cancer Genome Atlas (TCGA) project dataset is a comprehensive atlas of gene expression and regulation across human cancers, which has been used to assess gene expression levels in patients with head and neck squamous cell carcinoma (HNSCC).^21^ HPV infection status was obtained through the University of California Santa Cruz (UCSC)‐Xena database using HPV in situ hybridization and/or p16 expression as indicators of HPV infection.^22^ Differentially expressed genes (DEGs) were screened according to the HPV infection status. R CRAN ‘ggplot2’ and ‘ggsignif’ libraries were used to plot and estimate Pearson correlation coefficients and *p*‐values, respectively.^23^, ^24^

### 2.3 RNA isolation and quantitative real‐time polymerase chain reaction (qRT‐PCR)

After flushing the medium with PBS, 1 mL of Trizol was added to the cells and reacted for 5 min at room temperature. The total RNA was extracted by phenol/chloroform extraction and isopropanol precipitated. RNA concentration and purity were measured using a Spark multimode microplate reader (Tecan, Männedorf, Switzerland). The DNase was applied to remove genomic DNA, and then the reverse transcription kit (TransGen Biotech) and random primers were utilized to synthesize the cDNA. Quantitative PCR was performed using a Rotor‐Gene Q real‐time PCR cycler (Qiagen, Hilden, Germany) and Power SYBRs Green PCR Master Mix (Qiagen). The primers were purchased from Kumei Biotechnology Co. (Jilin, China) and the primer sequences are listed in Table S4. With each sample, there was one replicate omitting the reverse transcription step performed as the negative control. β‐actin was used as an internal control. Data were presented as the average of each replicate normalized to the average of β‐actin (±SEM).^25^

### 2.4 Statistical analysis and establishment of nomogram model

Statistical analyses were performed using SPSS version 26.0 (IBM Corp., Armonk, NY, USA). Categorical data were reported as frequencies and percentages (%), and continuous data were expressed as means ± standard deviations (SD). The prognostic factors for PFS and OS were analysed using Kaplan–Meier curves. Univariable and multivariable Cox proportional hazards regression were used to evaluate factors associated with PFS and OS. Factors that were significant in the univariable Cox proportional hazards regression were added to the multivariable Cox proportional hazards ratio model. The T and N stages were excluded from the multivariable model because of multicollinearity.

In addition, we constructed the Nomogram to predict the prognosis of OPSCC based on independent prognostic factors. Nomogram models were meticulously crafted employing R software version 4.0.5 and harnessing the power of R packages, ‘survival’ (version 3.4–0), ‘survminer’ (version 0.4.9) and the ‘timeROC’ (version 0.4). Univariate Cox proportional hazard regression analysis was carried out to scrutinize the top 20 genes exhibiting distinct expression patterns among HPV‐positive, and HPV‐negative cases. Variables (Including clinical features and DEGs) with a significance level of *p* < 0.05 were subsequently integrated into a multivariate Cox proportional hazard regression analysis to identify independent prognostic factors for TCGA‐HNSC. Following this, two nomogram model plots were generated based on these independent prognostic factors, encompassing OS and PFS, for predicting the prognosis associated with HNSC. The accuracy of the nomograms was evaluated using the C‐index and receiver operating characteristic (ROC) analysis, and the nomogram's discriminative ability was confirmed through the calibration plots. Decision curve analysis (DCA) was employed as a method to evaluate clinical prediction models by considering the potential range of patient risks and benefits.^26^ DCA assesses the practicality of clinical decisions by analysing the model's capacity to predict outcomes. The advantage of this approach is that it compensates for the limitations of ROC curves by displaying false positive and true positive rates as functions of risk thresholds.

## 3 RESULTS

### 3.1 Patient characteristics

Based on the inclusion and exclusion criteria, the records of 233 patients with histologically confirmed OPSCC were retrospectively analysed. The patients' baseline characteristics are shown in Table S1. Of the 233 patients, 207 (88.8%) were male and 26 (11.2%) were female. Their average age was 58.4 years. Most patients had a history of smoking (63.5%) and alcohol consumption (65.4%). The tonsils (37.8%) and root of the tongue (41.3%) were the most common primary tumour sites. The most common grades of pathological differentiation were moderately differentiated (55.1%) and well differentiated (16.2%). Most patients had lymphovascular invasion, and approximately 50% had nerve invasion. The p16‐IHC results showed that 55 patients were HPV positive and 48 patients were HPV negative. More than 50% of the patients were diagnosed with locally advanced disease (stage III: 26.3%, stage IV: 56.3%), and only 17.4% had early‐stage (stages I and II) disease.

### 3.2 Prognostic factors affecting progression‐free survival

**FIGURE 1:**
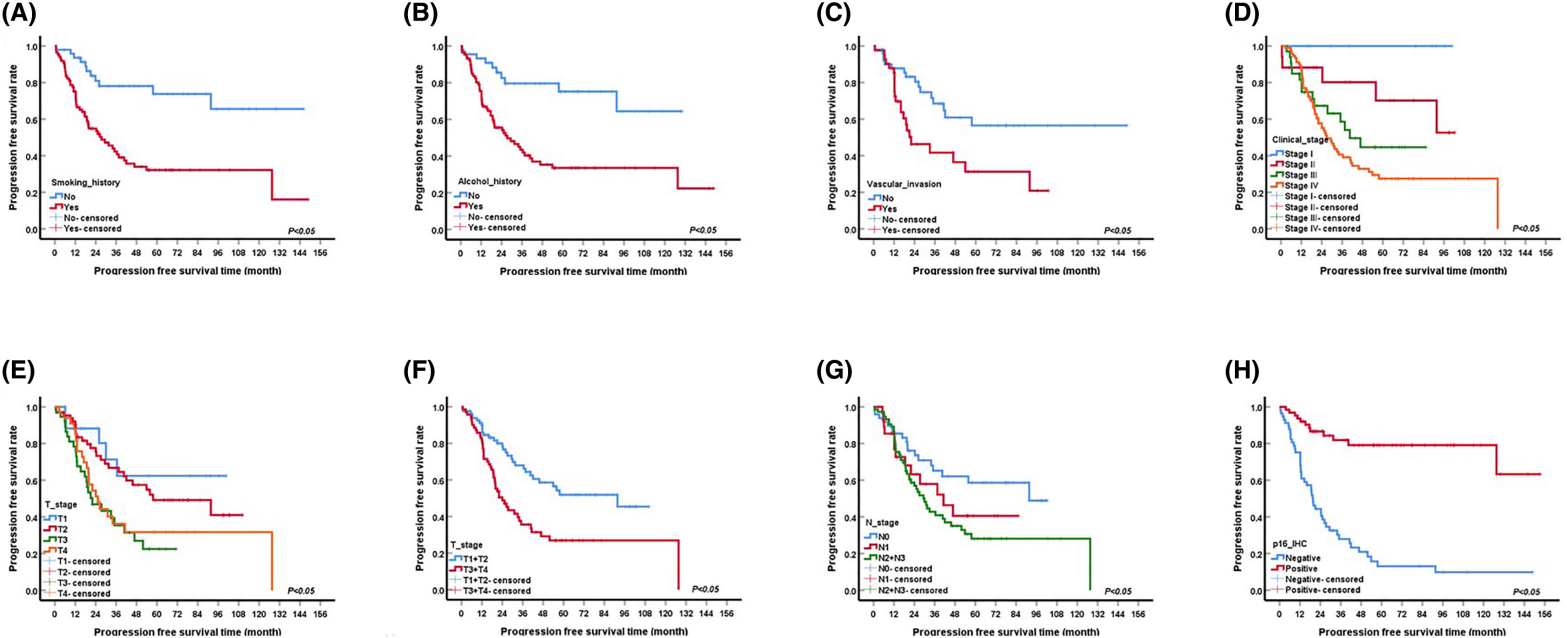
Kaplan–Meier curves showing the progression‐free survival of oropharyngeal squamous cell carcinoma by univariate analysis, including significantly different results (*p* < 0.005). (A) Tobacco. (B) Alcohol. (C) Lymphovascular invasion. (D) Clinical stage. (E) T stage. (F) T stage (Regrouped). (G) N stage. (H) p16‐IHC.

The median PFS survival was 37.6 months (Figure S1A). The univariable analysis showed that smoking, alcohol consumption, lymphovascular invasion, T/N stage, clinical stage and HPV infection were significantly associated with PFS (Table S2 and Figure 1).

The Kaplan–Meier analysis showed that patients with a history of smoking and alcohol consumption were more susceptible to recurrence than patients without a history of smoking and alcohol consumption (*p* < 0.001) (Figure 1A,B). The 1‐, 3‐ and 5‐year PFS of patients without lymphovascular invasion were 87.8%, 68.5% and 56.6%, respectively, which were significantly better than those with lymphovascular invasion (*p* = 0.006) (Figure 1C). The 1‐,3‐,5‐ year PFS for patients with locally advanced disease were 82.9%, 35.6% and 26.9%, respectively, which were significantly poorer than those patients with T1/T2 stage (Figure 1D–G). Patients with HPV‐positive tumours had significantly better PFS than in those patients with HPV‐negative tumours (*p* < 0.001) (Figure 1H). However, there was no significant difference in PFS between patients of different genders, ages and tumour sites, and the presence of nerve or marginal invasion (Figure S2).

The results of the multivariable Cox regression analysis of the factors associated with PFS were shown in Table S3. The multivariable Cox regression analysis showed that HPV infection and alcohol consumption were independent risk factors for PFS, which was lower in HPV‐negative patients than in HPV‐positive patients (HR: 7.93, 95% CI: 2.38–26.44, *p* = 0.001).

### 3.3 Prognostic factors affecting overall survival

**FIGURE 2:**
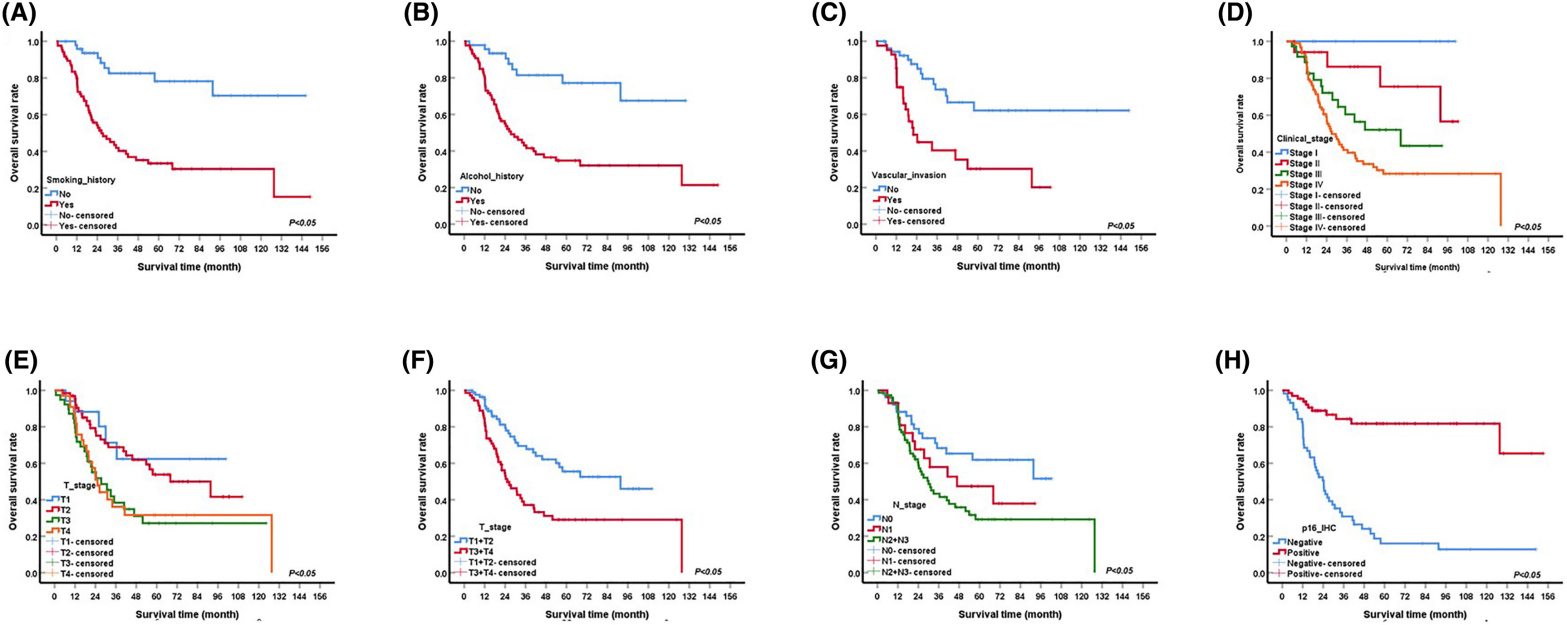
Kaplan–Meier curves showing the overall survival (OS) of oropharyngeal squamous cell carcinoma by univariate analysis, including significantly different results (*p* < 0.005). (A) Tobacco. (B) Alcohol. (C) Lymphovascular invasion. (D) Clinical stage. (E) T stage. (F) T stage (Regrouped). (G) N stage. (H) p16‐IHC.

The median survival time of OS was 42.0 months (Figure S1B). The results of the univariable analysis showed that smoking, alcohol consumption, lymphovascular invasion, T/N stage of tumour, clinical stage and HPV infection were significantly associated with OS (Table S2 and Figure 2).

Kaplan–Meier curves showed that the median survival time of patients who smoked was 26.4 months, and the 1‐, 3‐, and 5‐year OS were 79.8%, 41.9% and 33.5%, respectively, which were significantly lower than those of non‐smoking patients (*p* < 0.001) (Figure 2A). The 1‐, 3‐, and 5‐year OS of patients without a history of alcohol consumption were 97.9%, 81.4% and 77.2%, respectively, which were significantly higher than those of patients who consumed alcohol (Figure 2B). The median survival time of patients with lymphovascular invasion was 22 months, which was significantly lower than that of patients without lymphovascular invasion (*p* < 0.001) (Figure 2C). In addition, we compared patients with locally early‐stage disease (T1/T2) with those with locally advanced‐stage disease (T3/T4) and found that the 1‐, 3‐, and 5‐year survival of patients with locally early‐stage disease were 96.4%, 69.5% and 55.5%, respectively, which were significantly higher than those of patients with locally advanced‐stage disease (Figure 2D–F). Similar results were obtained for N stage: patients without lymph node metastasis had a significantly higher OS than those with N2 and N3 disease (*p* = 0.005) (Figure 2G). Most importantly, HPV infection was confirmed to be a good prognostic factor for OS. The 1‐, 3‐, and 5‐year survival of HPV‐positive patients were 95.5%, 84.3% and 81.8%, respectively, which were significantly higher than those of HPV‐negative patients (80.8%, 30.9% and 16.0%, respectively) (Figure 2H). Moreover, there was no significant difference in OS between patients of different gender, age, tumour site and with or without nerve or marginal invasion (Figure S3).

Alcohol consumption and HPV infection status were included in the multivariable Cox regression model (Table S3), but the T and N stages were excluded because of multicollinearity. The results showed that HPV infection was an independent prognostic factor for OS. Compared with HPV‐negative patients, HPV positive patients had a significantly lower risk (HR: 0.14, 95% CI: 0.04–0.47, *p* = 0.001).

### 3.4 Identification of differentially expressed genes in HPV‐positive and ‐negative OPSCC

To further explore the critical molecules of HPV infection affecting the prognosis of OPSCC, we explored and validated the genomic data of HPV‐positive and HPV‐negative OPSCC samples from the TCGA database. The TCGA data showed that 260 genes were upregulated and 470 genes were downregulated in the HPV‐positive group compared to the HPV‐negative group. The genes were enriched in terms of biological processes, cellular components and molecular functions (Figure S4A–C). The DEGs were closely associated with extracellular matrix (ECM) organization, epidermal cell differentiation and other biological processes. In addition, most DEGs were encoded by ECM components, or endoplasmic reticulum components. The DEGs were involved primarily in the function of glycosaminoglycan, which are the ECM structural constituent. Kyoto Encyclopedia of Genes and Genomes (KEGG) pathway enrichment analysis showed that the DEGs were mainly enriched in the PI3K‐Akt signalling pathway, focal adhesion and HPV infection pathway (Figure S4D–F).

**FIGURE 3:**
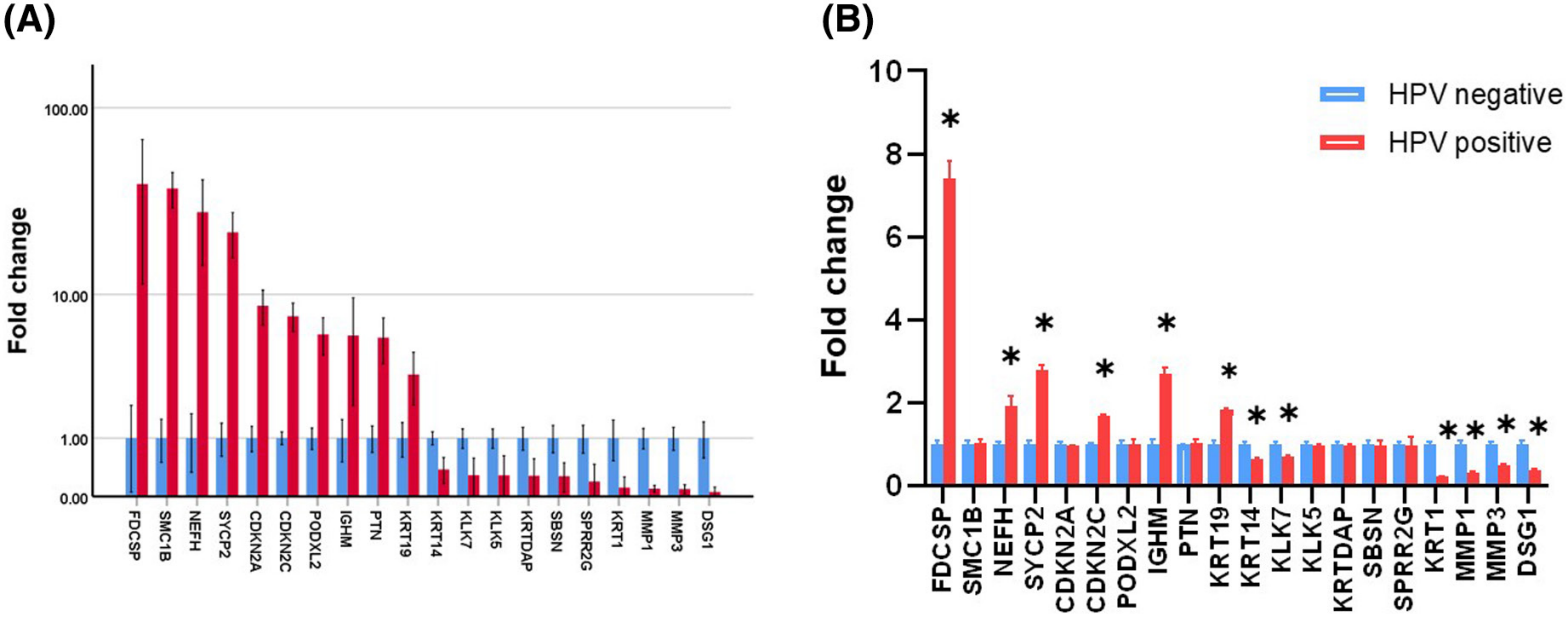
(A) The level of the differential gene was summarized from TCGA database. (B) The mRNA level of the differential gene was detected by quantitative real‐time polymerase chain reaction. *p < 0.05 versus HPV negative.

We selected the 10 upregulated and 10 downregulated genes with the largest difference (Figure 3A). According to the genetic central dogma, genes undergo transcription to mRNA and translation into proteins to exert their effects. Therefore, we examined the mRNA expression of the DEGs. The results showed that, the mRNA levels of *FDCSP*, *KRT19*, *IGHM*, *CDKN2C* and *SYCP2* were significantly upregulated and those of *MMP1*, *MMP3*, *KRT14* and *KLK7* were significantly downregulated in HPV‐positive group compared with the HPV‐negative group (Figure 3B).

### 3.5 Prognostic nomogram model

**FIGURE 4:**
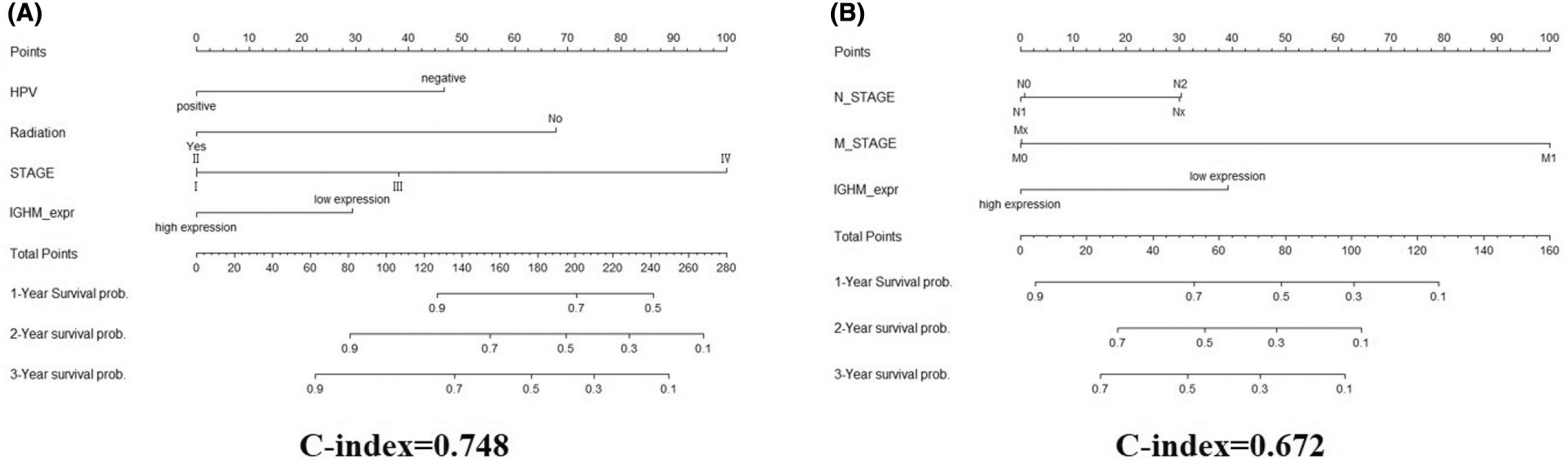
Nomogram was used to predict overall survival (OS) of patients with head and neck squamous cell carcinoma from TCGA database. To use this nomogram, the patient's specific point for each variable is located on each variable axis. A vertical line was drawn upward to determine the points for each variable; th4.e sum of these points was located on the total points line, and a vertical line is drawn downward to the survival axis to determine the probability of at OS 1, 2 and 3 years. (A) Nomoplot of OS. (B) Nomoplot of progression free survival.

We performed a univariate Cox regression on factors within the clinical features on patients in TCGA‐HNSC. The results revealed that HPV infection, radiotherapy and clinical staging were three independent predictors for OS. Additionally, gene expression of *IGHM* among the previously screened HPV‐DEGs was also identified as an independent predictor for the OS survival of HNSCC patients. When conducting univariate Cox regression for PFS, N staging, M staging and *IGHM* expression were found to be independent predictors for PFS in TCGA‐HNSC patients. Subsequently, based on the independent predictors selected from our univariate Cox model, we constructed nomogram models for TCGA‐HNSC (Figure 4).

**FIGURE 5:**
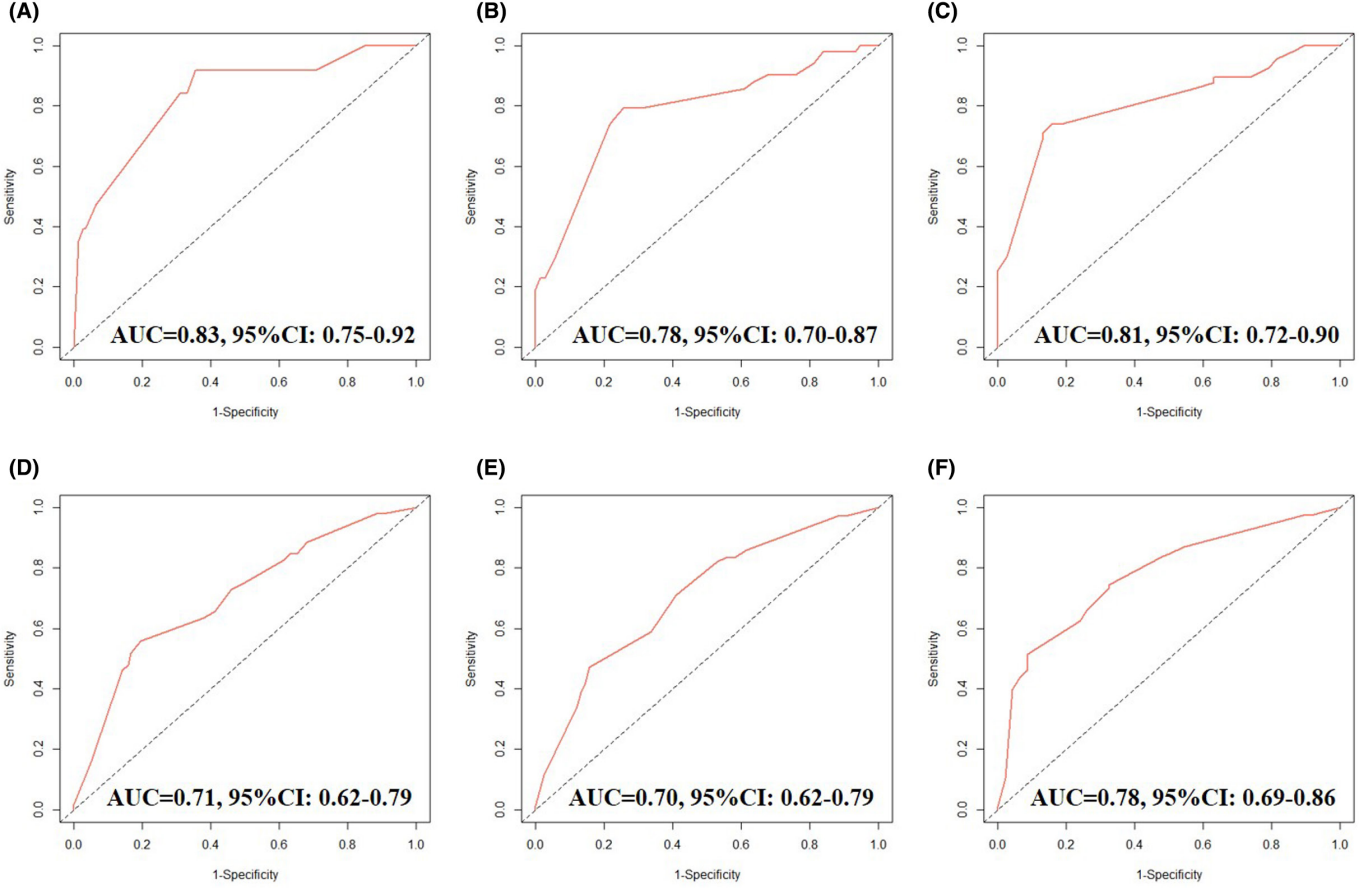
Calibration curves of the nomograms. Calibration curves of 1‐, 2‐ and 3‐year overall survival (A–C) and progression free survival (D–F) for head and neck squamous cell carcinoma patients. The dotted line represents the ideal reference line, where the predicted probability would match the observed survival rate. The red dots are calculated by bootstrapping (resample: 50) and represent the nomogram performance. The closer the solid red line is to the dotted line, the more accurate the model is in predicting OS.

**FIGURE 6:**
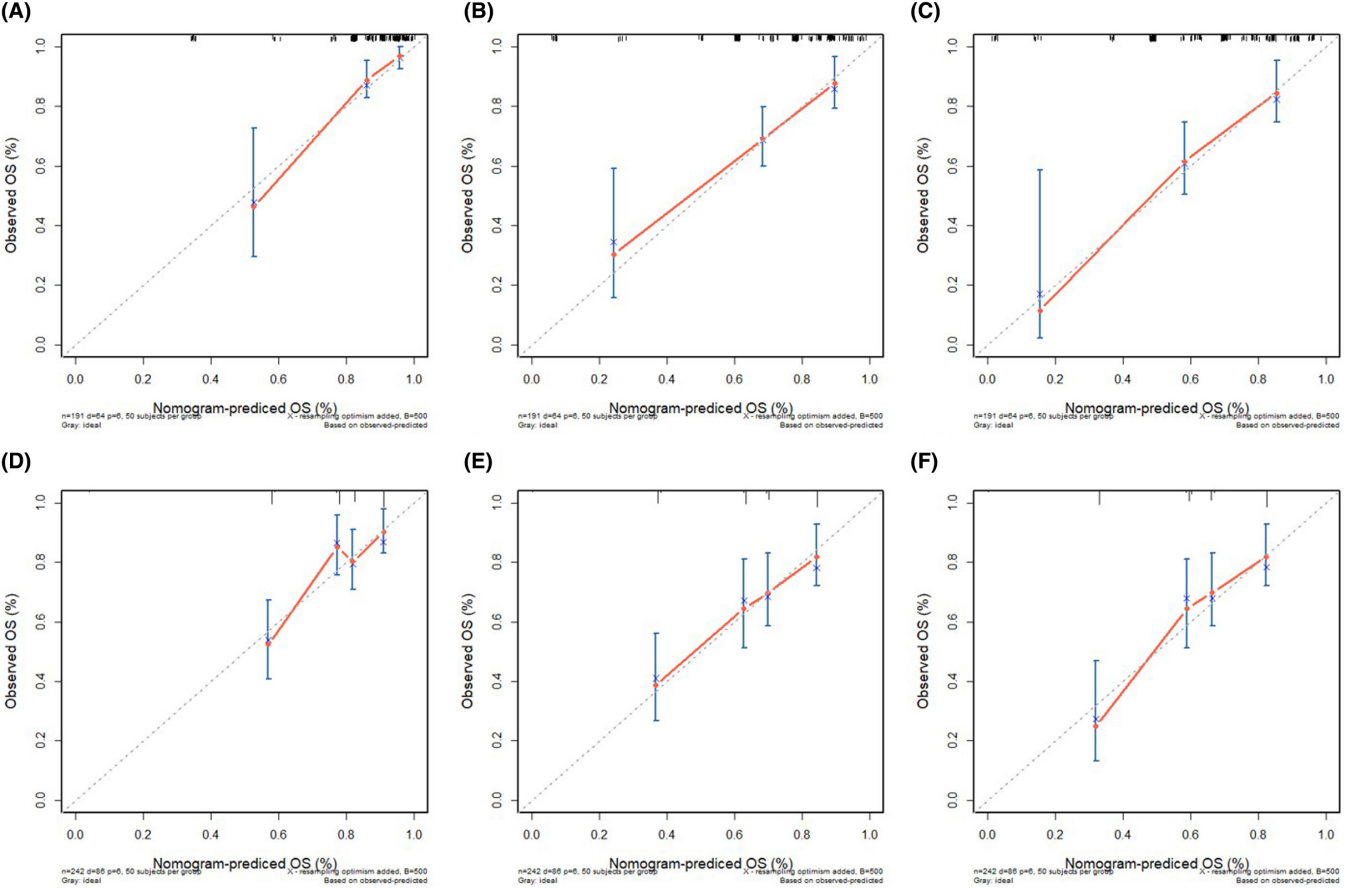
Receiver operating characteristic curves of the ability of nomogram to predict 1‐, 2‐ and 3‐year overall survival (A–C), progression free survival (D–F).

**FIGURE 7:**
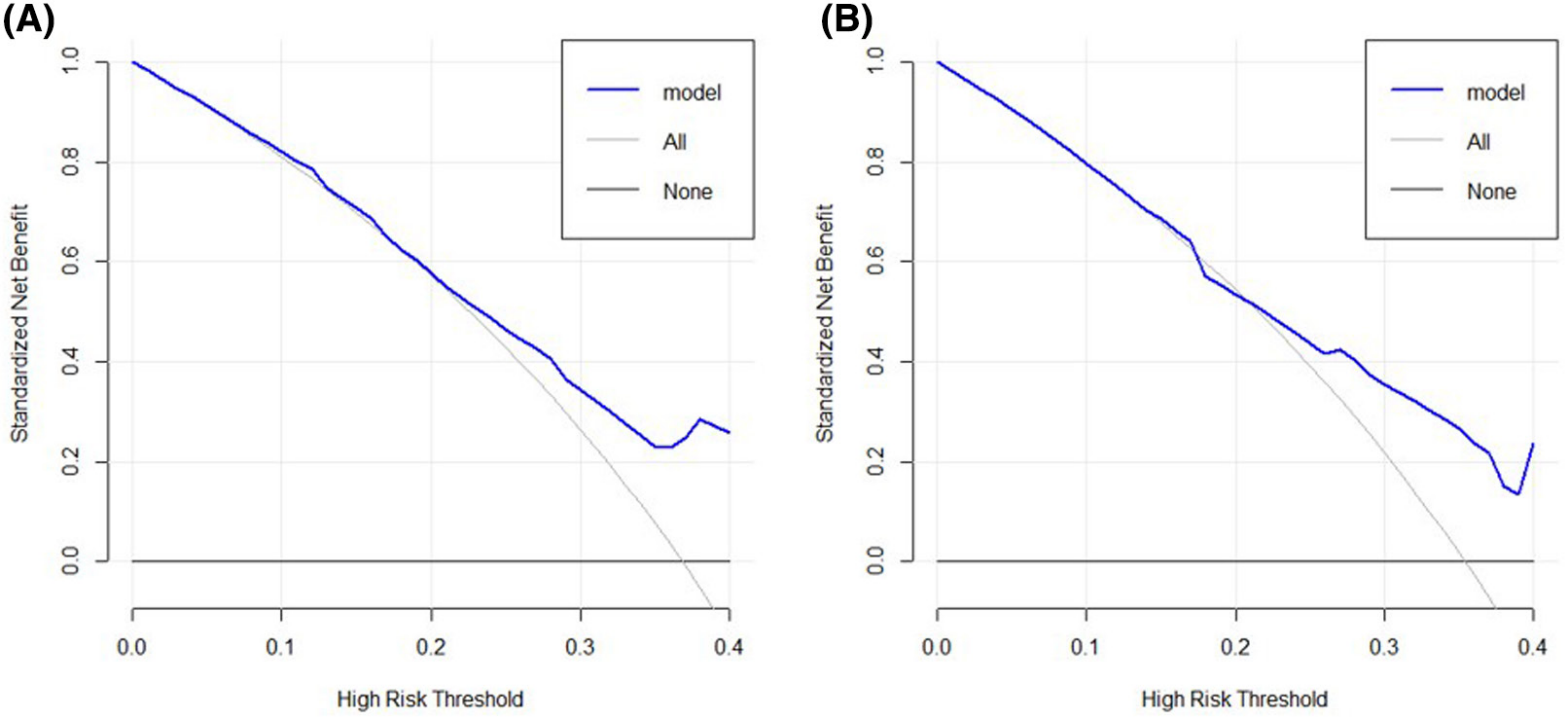
Decision curve analysis of the nomogram for survival prediction of head and neck squamous cell carcinoma patients. (A) Survival benefit for OS; (B) Survival benefit for PFS.

The results of the calibration curve were consistent with the predictive outcomes, with the solid red line closely following the dotted line, indicating a higher accuracy in predicting OS (Figure 5). ROC analysis demonstrated Area Under Curve (AUC) values of 1‐, 2‐ and 3‐year OS were 0.83, 0.78 and 0.8, and AUC values of 1‐, 2‐ and 3‐year PFS were 0.71, 0.70 and 0.78, respectively, suggesting the model's strong discriminative capacity (Figure 6). Furthermore, DCA also indicated the effectiveness of the nomogram model in clinical practice (Figure 7).

## DISCUSSION

First, we demonstrated that HPV infection exerted an essential role in the prognosis of OPSCC patients by analysing 233 OPSCC patients from our center. However, the underlying mechanisms remain inconclusive. Next, we analysed the DEGs in HPV‐infected and non‐infected groups in the TCGA database. We identified 20 DEGs and subjected them to Cox regression analysis. Our study, for the first time, established a nomogram model incorporating *IGHM* expression to predict the prognosis of HNSC patients. ROC curves, calibration plots and DCA curves indicated the model's predictive capacity. Our nomogram provides valuable guidance for clinical practice and treatment adjustment.

Adverse lifestyle habits of patients, such as smoking and alcohol consumption, are important factors in the development of OPSCC and have also been reported to affect patients' survival.^27^ This is consistent with the results of our previous studies on head and neck cancer.^28^ However, other studies reported contradictory results. França et al.^29^ reported that smoking and drinking have no effect on OS and PFS in patients with OPSCC. In this study, univariable analysis showed that patients with a history of smoking and alcohol consumption had a poorer median OS and PFS, suggesting that tobacco and alcohol consumption contribute to the poor prognosis in patients with OPSCC. In addition, gender has been reported to be prognostic factor, with female patients having a significantly better prognosis than male patients, even when the HPV status of the tumour was considered.^30^ However, previous results have shown contradictory results.^31^ The results of this study showed no statistically significant difference in OS or PFS in gender, which may be due to the predominantly male patient sample. Tumour stage and vascular invasion also significantly influenced survival; however, there were no significant differences in survival according to invasion of the surgical margins or nerves. T‐stage and N‐stage are well‐recognized factors affecting the prognosis of patients with OPSCC. Consistent with previous findings,^32^ in our study, OS and PFS were significantly lower in patients with a more advanced T stage, N stage, vascular invasion and clinical stages III and IV than those in earlier stages. In addition, as few patients with N3 tumours were included in our study, we combined N2 and N3 results. Based on the IHC results, we identified p16, a marker of HPV infection, as a factor affecting patient survival. In this study, patients who were p16 positive had significantly better survival than p16 negative patients, suggesting that HPV‐positive tumours may be more responsive to treatment in patients with OPSCC. Currently, there is no consistent conclusion on the role of HPV in influencing the prognosis of HNSCC. A meta‐analysis has confirmed that HPV‐positive OPSCC patients had a 52%, 63% and 53% reduction in tumour progression, recurrence and mortality, respectively, compared with HPV‐negative patients.^33^ On the contrary, it has been reported in some studies that HPV infection with types other than HPV16 may be associated with poor prognosis of OPSCC.^8^, ^34^ At the same time, there are also studies suggesting that HPV infection has no significant effect on the prognosis of OPSCC beyond the tonsil and tongue root sites.^35^, ^36^ This suggests that the relationship between HPV and the HNSCC prognosis deserves to be emphasized, which needs to be demonstrated in further large clinical studies. In addition to health promotion, we focused specifically on the benefits of HPV infection. HPV is well known to be an important causative factor of cervical cancer.^37^ However, in OPSCC, HPV infection has been identified to be associated with a better prognosis.^38^, ^39^ Nevertheless, the mechanisms by which HPV infection improves the prognosis and responsiveness to treatment in patients with OPSCC have not been reported in detail.

To screen for molecular markers of HPV infection affecting OPSCC prognosis, we searched the TCGA database for data from HPV‐positive and HPV‐negative patients with OPSCC. The gene expression of HPV‐positive and HPV‐negative patients differed significantly. Further analysis revealed that the DEGs in the two groups were mainly related to epidermal development, external encapsulating structure organization and ECM organization. These functions are mainly responsible for tumour growth, invasion and metastasis, and are regulated by multiple mechanisms. Furthermore, we enriched HPV‐positive and HPV‐negative DEGs in the KEGG pathway. The results suggested that the DEGs were mainly concentrated in focal adhesion, ECM‐receptor interaction, and the advanced glycation end‐products (AGE)‐receptor for AGE (RAGE) (AGE‐RAGE) signalling pathway. Based on the fold difference of the genes, we identified the 10 most significantly upregulated DEGs and the 10 most significantly downregulated DEGs.

Further validation of mRNA levels using quantitative real‐time PCR revealed that *FDCSP*, *KRT19*, *IGHM*, *CDKN2C* and *SYCP2* mRNA levels were upregulated and *MMP1*, *MMP3*, *KRT14* and *KLK7* mRNA levels were downregulated in the HPV‐positive group compared to those in the HPV‐negative group. These DEGs were mainly associated with the proliferation, differentiation and immune characteristics of squamous cancer cells. This suggests that HPV infection may influence prognosis by altering cell proliferation, differentiation and the immune microenvironment.

In the OS nomogram, HPV infection and radiotherapy had an impact on patient survival, but this effect was not observed in PFS. We speculate that since HNSC patients generally have a favourable overall prognosis, the influence of HPV infection and radiotherapy on survival may outweigh their impact on tumour progression, which explains their value was not evident in the model. Surprisingly, *IGHM* had a significant negative effect on both OS and PFS, suggesting that *IGHM* may play a crucial negative role in prognosis, and warrants further experimental validation. *IGHM* is an immune‐related gene which has been shown to be associated with immune response^40^ and prognosis^41^, ^42^ in breast cancer. Furthermore, *IGHM* has been also reported to impact the recurrence of multiple myeloma^43^ and the progression pathways of follicular lymphoma.^44^ However, the correlation between *IGHM* expression and HPV infection and the influence of IGHM on the prognosis of HNSCC have not been reported. The identification of this key molecule could hopefully provide a new molecular target for the treatment of HNSCC.

The main limitation of this study is that the patient data were derived from a single center, and the sample size was relatively small. In addition, M staging was not included in this study, because most patients had access to surgery. In this study, we established a relatively robust evaluation system to assess the prognosis of HNSC patients. We visualized our results with nomograms to assist clinicians in making better decisions. Although we used large‐scale sequencing data from TCGA for analysis and model development, and evaluated the model, it is important to note that TCGA data primarily represent a specific racial population (mostly Caucasian), and therefore it can be inferred that the model may have limited ability to predict the prognosis of HNSC in other racial groups may be limited. Additionally, we did not provide external validation for the model, which imposes some limitations on its reliability. Furthermore, all data used in our study were retrospective, and therefore causal effects cannot be inferred. Prospective cohort studies or experimental research are needed to confirm our findings. Given that prognosis in patients is influenced by multiple factors (nutritional status, specific medications and treatment regimens), it appears challenging to identify all the influencing factors, and impossible to assess the impact of these factors on the variables in the model.

In this study, we confirmed that HPV infection is an independent prognostic factor for OPSCC by applying our center's data. In addition, we developed a prognostic prediction model of OPSCC and discovered *IGHM* as a key molecule. Nomogram model provides valuable guidance for clinical practice and treatment adjustment.

## AUTHOR CONTRIBUTIONS

**Xin Jiang:** Conceptualization (equal); funding acquisition (equal); validation (equal); writing – review and editing (equal). **Huanhuan Wang:** Data curation (equal); formal analysis (equal); investigation (equal); writing – original draft (equal). **Qihe Zhang:** Conceptualization (equal); funding acquisition (equal); validation (equal); writing – review and editing (equal). **Zhuangzhuang Zheng:** Investigation (equal); writing – original draft (equal). **Ying Xin:** Conceptualization (equal); funding acquisition (equal); writing – review and editing (equal).

## FUNDING INFORMATION

This research was funded by the Jilin Provincial Science and Technology Foundation (grant number 220230508064RC), the Education Department Foundation of Jilin Province (Grant number JJKH20241328KJ), the Youth Development Foundations of First Hospital of Jilin University, grant number (Grant number JDYY14202316, JDYY‐DEP‐2022002).

## CONFLICT OF INTEREST STATEMENT

The authors declare that the research was conducted in the absence of any commercial or financial relationships that could be construed as a potential conflict of interest.

## Supporting information


Figure S1.

Figure S2.

Figure S3.

Figure S4.

Table S1.

## Data Availability

The original contributions presented in the study are included in the article/supplementary materials. Further inquiries can be directed to the corresponding authors.
