# Supplementary figures and images for: Differential molecular characterization of human papillomavirus‐associated oropharyngeal squamous cell carcinoma and its prognostic value
Source: J Cell Mol Med. 2024 Oct 13;28(19):e70073. doi: 10.1111/jcmm.70073 (PMC11471427; doi:10.1111/jcmm.70073)

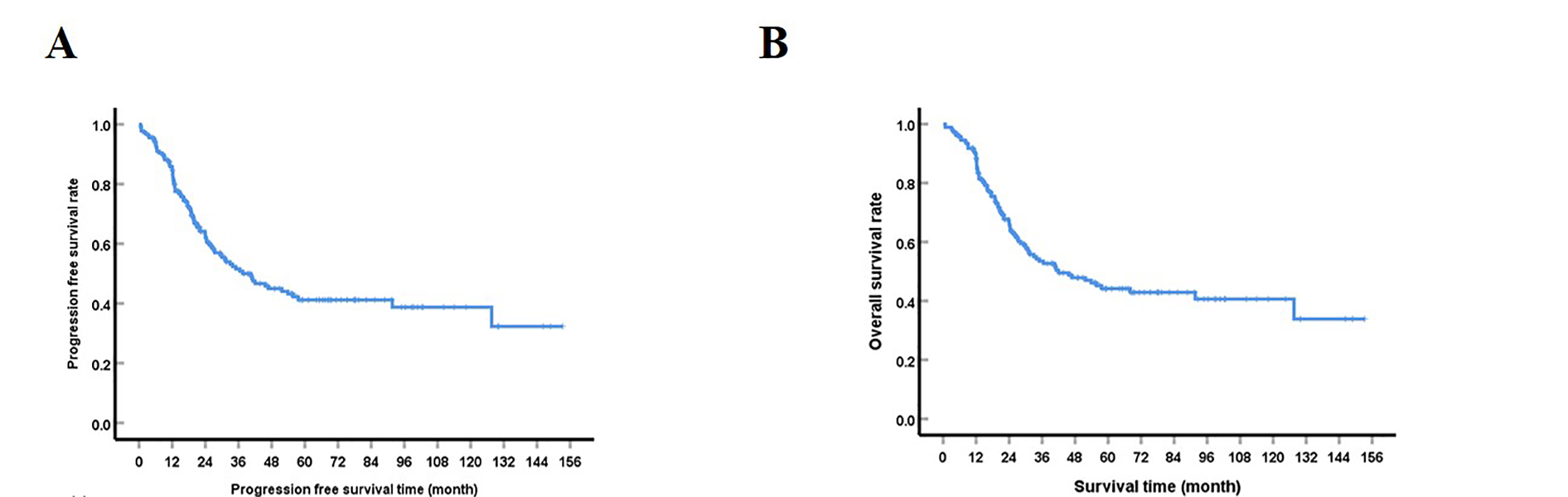

Supplement: Supplementary file 1 — Figure S1. [file JCMM-28-e70073-s002.jpg]

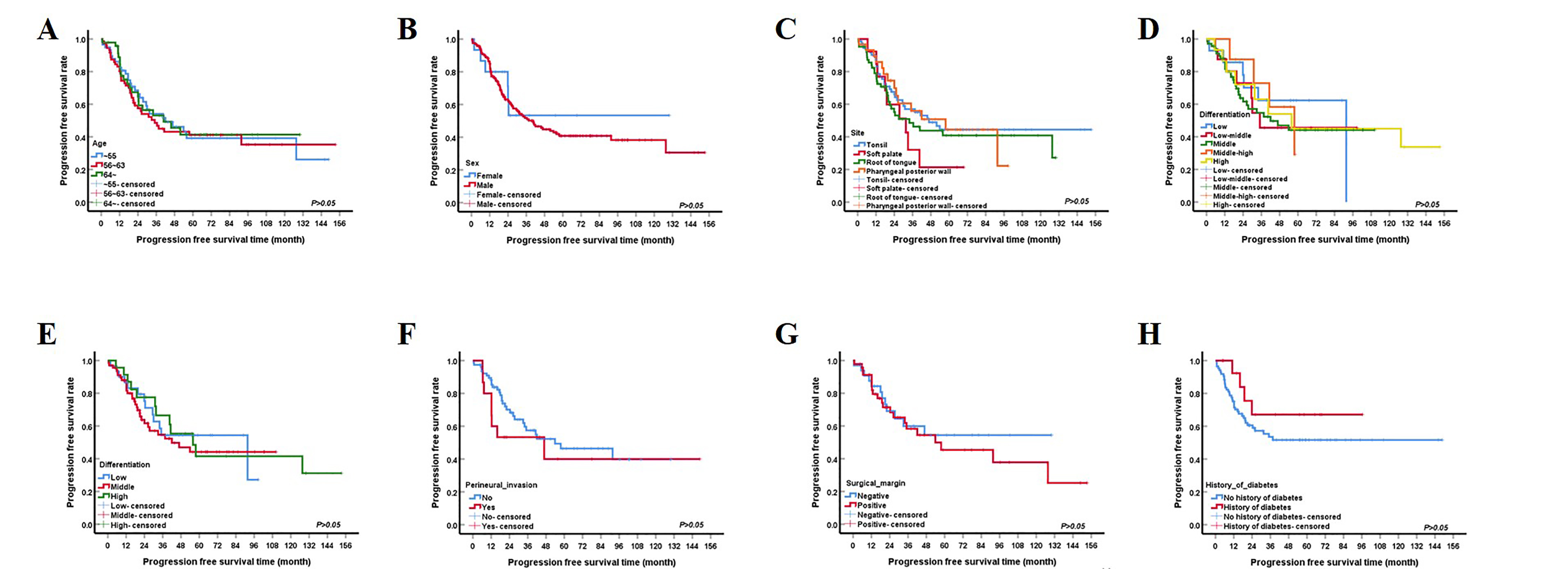

Supplement: Supplementary file 2 — Figure S2. [file JCMM-28-e70073-s001.jpg]

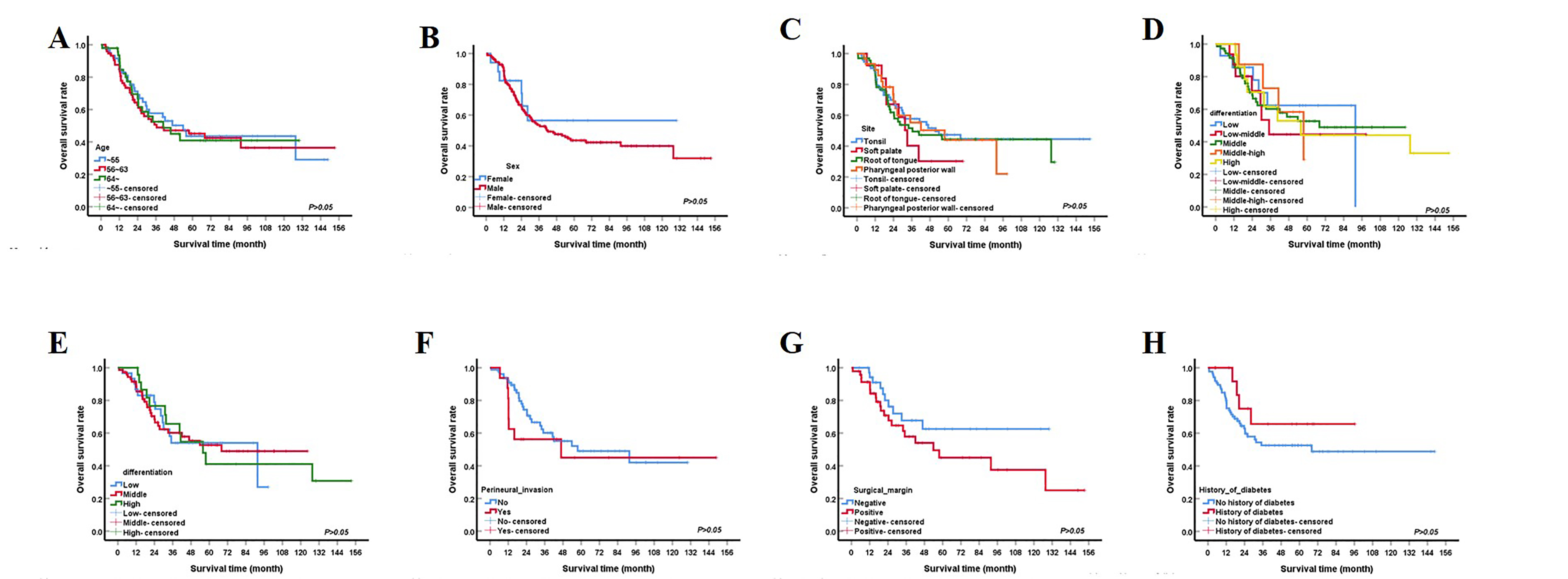

Supplement: Supplementary file 3 — Figure S3. [file JCMM-28-e70073-s004.jpg]

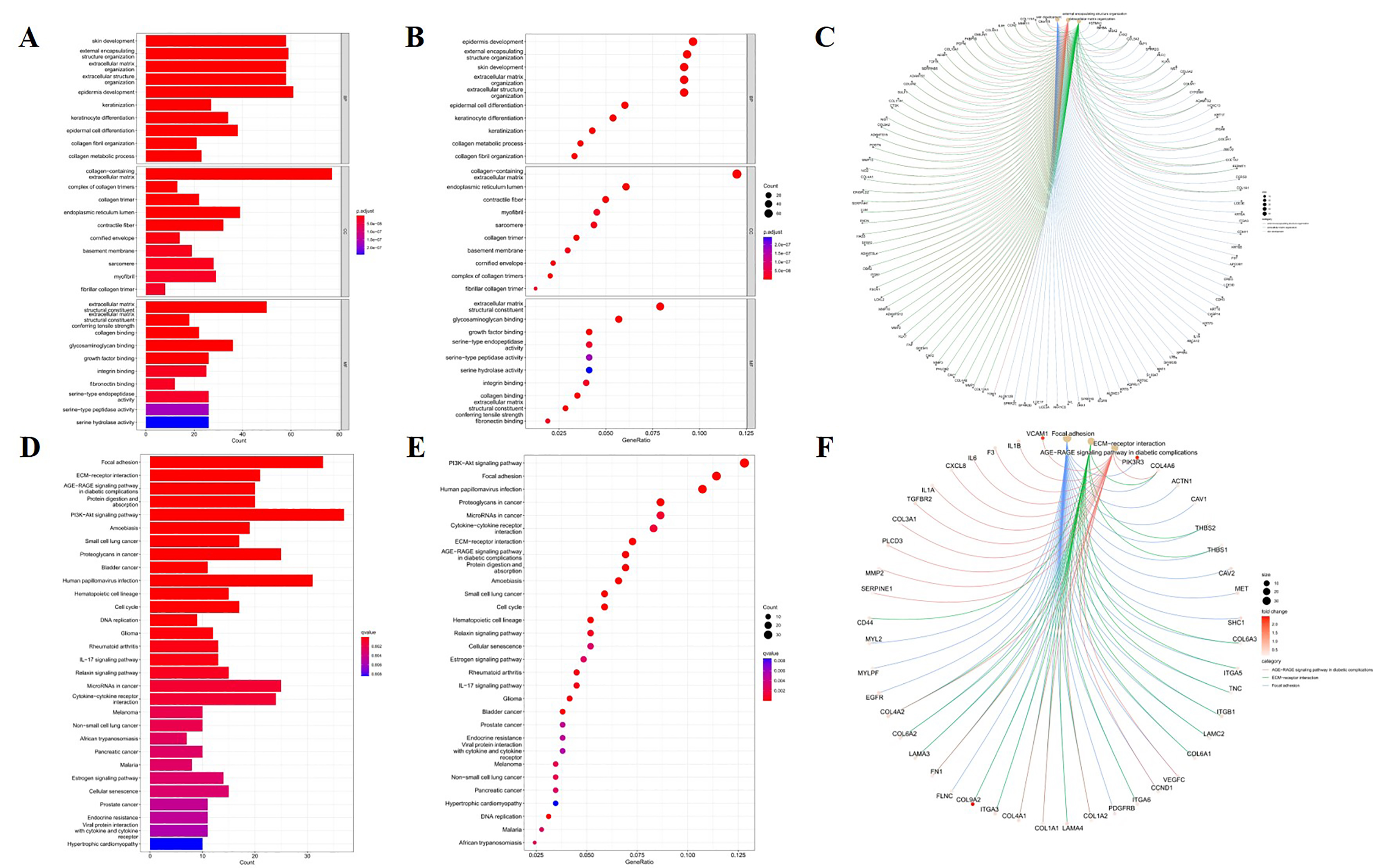

Supplement: Supplementary file 4 — Figure S4. [file JCMM-28-e70073-s005.jpg]
