# Supplementary material for: Differential molecular characterization of human papillomavirus‐associated oropharyngeal squamous cell carcinoma and its prognostic value
Source: J Cell Mol Med. 2024 Oct 13;28(19):e70073. doi: 10.1111/jcmm.70073 (PMC11471427; doi:10.1111/jcmm.70073)
Supplement: Supplementary file 5 — Table S1. [file JCMM-28-e70073-s003.docx]

**Supplementary table 1.** Summary of baseline characteristics.

| **Characteristics** | | **Frequency Percent/Mean ± SD** |
| --- | --- | --- |
| **Gender** | |  |
|  | Female | 26 (11.2%) |
|  | Male | 207 (88.8%) |
| **Age** | |  |
|  | Mean ± SD | 58.4 ± 9.36 |
| **Tobacco** | |  |
|  | No | 50 (36.5%) |
|  | Yes | 87 (63.5%) |
| **Alcohol** | |  |
|  | No | 47 (34.6%) |
|  | Yes | 89 (65.4%) |
| **Site** | |  |
|  | Tonsil | 87 (37.8%) |
|  | Soft palate | 14 (6.1%) |
|  | Tongue root | 95 (41.3%) |
|  | Pharyngeal posterior wall | 34 (14.8%) |
| **Pathological differentiation** | |  |
|  | Low | 19 (11.4%) |
|  | Low-middle | 18 (10.8%) |
|  | Middle | 92 (55.1%) |
|  | Middle-high | 11 (6.6%) |
|  | High | 27 (16.2%) |
| **Lymphovascular invasion** | |  |
|  | No | 65 (58.0%) |
|  | Yes | 47 (42.0%) |
| **Perineural invasion** | |  |
|  | No | 94 (83.2%) |
|  | Yes | 19 (16.8%) |
| **Surgical margin** | |  |
|  | Negative | 48 (46.6%) |
|  | Positive | 55 (53.4%) |
| **p16-IHC** | |  |
|  | Negative | 63 (48.1%) |
|  | Positive | 68 (51.9%) |
| **T stage** | |  |
|  | T1 | 20 (11.9%) |
|  | T2 | 73 (43.5%) |
|  | T3 | 42 (25.0%) |
|  | T4 | 33 (19.6%) |
| **N stage** | |  |
|  | N0 | 55 (32.9%) |
|  | N1 | 36 (21.6%) |
|  | N2 | 72 (43.1%) |
|  | N3 | 4 (2.4%) |
| **Clinical stage** | |  |
|  | Stage Ⅰ | 10 (6.0%) |
|  | Stage Ⅱ | 19 (11.4%) |
|  | Stage Ⅲ | 44 (26.3%) |
|  | Stage Ⅳ | 94 (56.3%) |
| **History of diabetes** | |  |
|  | No | 87 (84.5%) |
|  | Yes | 16 (15.5%) |

**Supplementary table 2.** Survival rates and univariate analysis of Kaplan-Meier.

|  |  | **Progression-free survival** | | | | | **Overall survival** | | | | |
| --- | --- | --- | --- | --- | --- | --- | --- | --- | --- | --- | --- |
|  |  | **Survival rate (%)** | | | **Log Rank (χ2)** | **P value** | **Survival rate (%)** | | | **Log Rank (χ2)** | **P value** |
|  |  | **1 year** | **3 year** | **5 year** |  |  | **1 year** | **3 year** | **5 year** |  |  |
| **Gender** | |  |  |  | 0.230 | 0.632 |  |  |  | 0.428 | 0.513 |
|  | Female | 80.0 | 53.3 | 53.3 |  |  | 82.4 | 56.5 | 56.5 |  |  |
|  | Male | 86.2 | 51.8 | 40.8 |  |  | 90.2 | 53.5 | 43.6 |  |  |
| **Age** | |  |  |  | 0.309 | 0.857 |  |  |  | 0.266 | 0.876 |
|  | ~55 | 86.0 | 54.1 | 39.2 |  |  | 88.1 | 57.7 | 43.6 |  |  |
|  | 56~63 | 83.1 | 48.6 | 41.2 |  |  | 87.6 | 50.6 | 45.1 |  |  |
|  | 64~ | 88.9 | 53.1 | 41.4 |  |  | 93.5 | 52.5 | 40.9 |  |  |
| **Tobacco** | |  |  |  | 17.303 | **<0.001** |  |  |  | 23.296 | **<0.001** |
|  | No | 93.6 | 78.1 | 73.8 |  |  | 97.9 | 82.6 | 78.2 |  |  |
|  | Yes | 75.0 | 42.3 | 32.2 |  |  | 79.8 | 41.9 | 33.5 |  |  |
| **Alcohol** | |  |  |  | 15.324 | **<0.001** |  |  |  | 19.052 | **<0.001** |
|  | No | 93.3 | 79.6 | 75.2 |  |  | 97.9 | 81.4 | 77.2 |  |  |
|  | Yes | 75.4 | 43.5 | 33.6 |  |  | 80.1 | 43.0 | 34.8 |  |  |
| **Site** | |  |  |  | 2.177 | 0.537 |  |  |  | 0.675 | 0.879 |
|  | Tonsil | 88.8 | 57.0 | 44.4 |  |  | 89.1 | 57.7 | 47.4 |  |  |
|  | Soft palate | 92.3 | 32.1 | 21.4 |  |  | 92.3 | 40.3 | 30.2 |  |  |
|  | Tongue root | 79.0 | 48.6 | 40.9 |  |  | 87.7 | 51.6 | 44.4 |  |  |
|  | Pharyngeal posterior wall | 89.6 | 55.9 | 44.4 |  |  | 93.2 | 55.3 | 44.0 |  |  |
| **Pathological differentiation** | |  |  |  | 0.764 | 0.943 |  |  |  | 0.341 | 0.987 |
|  | Low | 85.7 | 62.3 | 62.3 |  |  | 85.7 | 62.3 | 62.3 |  |  |
|  | Low-middle | 87.5 | 45.6 | 45.6 |  |  | 87.5 | 44.6 | 44.6 |  |  |
|  | Middle | 88.0 | 54.7 | 44.2 |  |  | 90.0 | 60.2 | 52.7 |  |  |
|  | Middle-high | 100.0 | 72.9 | 29.2 |  |  | 100.0 | 72.9 | 29.2 |  |  |
|  | High | 86.7 | 63.0 | 45.0 |  |  | 100.0 | 61.7 | 44.1 |  |  |
| **Lymphovascular invasion** | |  |  |  | 7.627 | **0.006** |  |  |  | 12.667 | **<0.001** |
|  | No | 87.8 | 68.5 | 56.5 |  |  | 94.2 | 73.6 | 62.1 |  |  |
|  | Yes | 87.9 | 41.4 | 31.3 |  |  | 87.7 | 40.3 | 30.2 |  |  |
| **Perineural invasion** | |  |  |  | 0.841 | 0.359 |  |  |  | 0.816 | 0.366 |
|  | No | 89.5 | 57.5 | 46.5 |  |  | 93.6 | 60.2 | 49.0 |  |  |
|  | Yes | 80.0 | 53.3 | 40.0 |  |  | 81.3 | 56.3 | 45.0 |  |  |
| **Surgical margin** | |  |  |  | 0.417 | 0.518 |  |  |  | 2.333 | 0.127 |
|  | Negative | 87.6 | 59.9 | 54.4 |  |  | 97.1 | 67.7 | 62.5 |  |  |
|  | Positive | 91.2 | 58.3 | 45.4 |  |  | 91.2 | 57.9 | 45.0 |  |  |
| **p16_IHC** | |  |  |  | 44.091 | **<0.001** |  |  |  | 43.149 | **<0.001** |
|  | Negative | 75.2 | 27.9 | 13.1 |  |  | 80.8 | 30.9 | 16.0 |  |  |
|  | Positive | 93.6 | 81.9 | 79.1 |  |  | 95.5 | 84.3 | 81.8 |  |  |
| **T stage** | |  |  |  | 11.481 | **0.001** |  |  |  | 12.086 | **0.001** |
|  | T1+ T2 | 91.3 | 68.0 | 51.9 |  |  | 96.4 | 69.5 | 55.5 |  |  |
|  | T3+ T4 | 82.9 | 35.6 | 26.9 |  |  | 83.3 | 37.1 | 29.1 |  |  |
| **N stage** | |  |  |  | 6.438 | **0.040** |  |  |  | 8.424 | **0.015** |
|  | N0 | 85.4 | 65.2 | 58.6 |  |  | 88.2 | 68.3 | 61.9 |  |  |
|  | N1 | 85.4 | 57.9 | 40.6 |  |  | 93.0 | 57.9 | 47.4 |  |  |
|  | N2+ N3 | 89.1 | 42.7 | 28.1 |  |  | 90.6 | 43.3 | 29.2 |  |  |
| **Clinical stage** | |  |  |  | 13.759 | **0.003** |  |  |  | 17.044 | **0.001** |
|  | Stage Ⅰ | 100 | 100 | 100 |  |  | 100.0 | 100.0 | 100.0 |  |  |
|  | Stage Ⅱ | 88.2 | 80.2 | 70.2 |  |  | 94.1 | 86.3 | 75.5 |  |  |
|  | Stage Ⅲ | 81.6 | 58.6 | 44.6 |  |  | 88.7 | 60.5 | 52.1 |  |  |
|  | Stage Ⅳ | 89.0 | 40.6 | 27.4 |  |  | 90.2 | 41.2 | 28.3 |  |  |
| **History of diabetes** | |  |  |  | 1.349 | 0.245 |  |  |  | 1.388 | 0.239 |
|  | No | 75.1 | 53.5 | 51.7 |  |  | 80.0 | 52.6 | 52.6 |  |  |
|  | Yes | 92.3 | 67.1 | 67.1 |  |  | 100.0 | 65.6 | 65.6 |  |  |

**Supplementary table 3.** Univariate/multivariable cox analysis of progression-free survival and overall survival.

|  |  | **Progression-free survival** | | | | **Overall survival** | | | |
| --- | --- | --- | --- | --- | --- | --- | --- | --- | --- |
|  |  | **Univariate Cox regression** | | **Multivariable**  **Cox regression** | | **Univariate Cox regression** | | **Multivariable Cox regression** | |
|  |  | **HR (95%Cl)** | **P value** | **HR (95%Cl)** | **P value** | **HR (95%Cl)** | **P value** | **HR (95%Cl)** | **P value** |
| **Tobacco** | |  |  |  |  |  |  |  |  |
|  | No | - |  |  |  | - |  |  |  |
|  | Yes | 3.66  (1.90, 7.04) | **<0.001** |  |  | 4.89  (2.40, 9.96) | **<0.001** |  |  |
| **Alcohol** | |  |  |  |  |  |  |  |  |
|  | No | - |  | - |  | - |  | - |  |
|  | Yes | 3.56  (1.81, 7.02) | **<0.001** | 3.67  (1.22, 11.05) | **0.021** | 4.27  (2.10, 8.68) | **<0.001** | 5.62  (1.63, 19.34) | **0.006** |
| **Lymphovascular invasion** | |  |  |  |  |  |  |  |  |
|  | No | - |  |  |  | - |  |  |  |
|  | Yes | 2.42  (1.27, 4.61) | **0.007** |  |  | 3.18  (1.63, 6.23) | **0.001** |  |  |
| **p16_IHC** | |  |  |  |  |  |  |  |  |
|  | Negative | 6.67  (3.51, 12.68) | **<0.001** | 7.93  (2.38, 26.44) | **0.001** | - |  | - |  |
|  | Positive | - |  | - |  | 0.14  (0.07, 0.28) | **<0.001** | 0.14  (0.04, 0.47) | **0.001** |
| **T stage** | |  |  |  |  |  |  |  |  |
|  | T1+ T2 | - |  |  |  | - |  |  |  |
|  | T3+ T4 | 2.17  (1.37, 3.44) | **0.001** |  |  | 2.22  (1.40, 3.53) | **0.001** |  |  |
| **N stage** | |  |  |  |  |  |  |  |  |
|  | N0 | - |  |  |  | - |  |  |  |
|  | N1 | 1.62  (0.79, 3.36) | 0.185 |  |  | 1.60  (0.78, 3.31) | 0.200 |  |  |
|  | N2+ N3 | 2.01  (1.16, 3.48) | **0.013** |  |  | 2.23  (1.28, 3.91) | **0.005** |  |  |
| **Clinical stage** | |  |  |  |  |  |  |  |  |
|  | Stage Ⅰ | - |  |  |  | - |  |  |  |
|  | Stage Ⅱ | 11473.49  (0.00, --) | 0.878 |  |  | 9273.24  (0.00, --) | 0.885 |  |  |
|  | Stage Ⅲ | 23565.52  (0.00, --) | 0.868 |  |  | 21603.31  (0.00, --) | 0.874 |  |  |
|  | Stage Ⅳ | 32473.97  (0.00, --) | 0.864 |  |  | 35162.63  (0.00, --) | 0.868 |  |  |

**Supplementary table 4.** Summary of specific primer sequences.

| Primer name | Forward primer | Reversed primer |
| --- | --- | --- |
| *FDCSP* | GCTGTTGGTTTCCCAGTCTCT | GTTGTAGGGGCAGATTCAGGT |
| *KRT19* | AGCAGGTCCGAGGTTACTGA | GCCGCTGGTACTCCTGATTC |
| *CDKN2A* | TGGGTTTGTAGAAGCAGGCAT | CCCAGGCATCTTTTGCACCT |
| *IGHM* | AGAAGTGAAAAAGCCGGGGG | GAGTCACCAGGATGGATGGTC |
| *CDKN2C* | CTCACACGGCTCAAGTCACC | CGCAGTCCTTCCAAATCCAT |
| *SYCP2* | CGGCTCCACCGACACTAC | GAACTGGGGGACTGGTTAGC |
| *PODXL2* | CTTCGGACCAACTCCCTCAC | TCCGGGCGGGTTTAATTGAG |
| *NEFH* | TCCAAAGGAGTGGTCAAGCC | AGGGAAAGAGAATAACAGCAGCA |
| *PTN* | GGAGAATGGCAGTGGAGTGT | CTTCCAGTTGCAGGGGATCT |
| *SMC1B* | GGTTCACCTGCATCATCGG | TTTCTCTTCGCCACTTTCCTCC |
| *SPRR2* | GAAGCTGGGAAGTAGGAGAAGC | CTAGTCCATGATCCTCCCCAG |
| *KRTDAP* | CGACCCGAGGCGTTTAAGG | GTTGCGCTCCTCAGTCCTTT |
| *MMP1* | AAGGCCAGTATGCACAGCTT | GGGCCACTATTTCTCCGCTT |
| *KLK5* | CATCTTTCTCTGTCCCCTCCTT | CATGGCCGCTGCACCTTATTT |
| *MMP3* | TGAGGACACCAGCATGAACC | CTTCCCCGTCACCTCCAATC |
| *DSG1* | ATCCAACCAACTTCCGGCAT | AGTTACGCCAGCACCAGAAA |
| *KRT14* | AGGAGATGATTGGCAGCGTG | TGCGATCCAGAGGAGAACTG |
| *KLK7* | TGCAGATCCTACTGCTATCCTT | ATTCATCTTGCAGTGGGCG |
| *SBSN* | AAGCGGATCTTCCAGCCATC | GATGAAAGGCGTGTTGACCG |
| *KRT1* | GATTGCCACCTACAGGACCC | ACAGACACACTCACGTTCGG |
